# A critical role of E2F transcription factor 2 in proinflammatory cytokines-dependent proliferation and invasiveness of fibroblast-like synoviocytes in rheumatoid Arthritis

**DOI:** 10.1038/s41598-018-20782-7

**Published:** 2018-02-08

**Authors:** Rui Zhang, Lin Wang, Ji-hong Pan, Jinxiang Han

**Affiliations:** 1 Shandong Medicinal Biotechnology Centre, Jinan, Shandong 250000 China; 2 Key Lab for Biotechnology Drugs of Ministry of Health, Jinan, Shandong 250000 China; 3 Key lab for rare & uncommon diseases, Jinan, Shandong 250000 China

## Abstract

As a transcription factor, E2F2 participates in regulation of numerous genes. To investigate the role and mechnism of E2F2 in RA, expression of E2F2 in synovial tissue was detected. Proliferation, invasion, and secretion of inflammatory cytokines were measured after E2F2 was knocked-down in RASFs by siRNA transfection. Induction of TNF-α, IL-6, and LPS on expression and nuclear translocation of E2F2, and signal pathways involved in the process were tested. ChIP was used to investigate direct binding of NF-кB to the promoter of E2F2, and E2F2 to the promoter of IL-6. The correlation between mRNA levels of E2F2 and IL-6 or TNF-α in secreted in supernatant of RASFs were also investigated. As a result, silencing E2F2 could inhibit the proliferation and invasion of RASFs. LPS, IL-6 can stimulate the expression of E2F2 in RASFs both via the NF-кB pathway, while TNF-α via the ERK pathway. TNF-α can facilitate the nuclear translocation of E2F2 and TNF-α can bind to promoter of E2F2, and then E2F2 can bind to the promoter of IL-6 directly. Significant correlations was found between levels of E2F2 and IL-6/TNF-α in synoviocytes of RA patients. Our findings indicate that E2F2 may play an important role in pathogenesis of RA.

## Introduction

Rheumatoid arthritis (RA) is a systemic autoimmune disease, characterized by hyperplastic synovial fibroblasts and progressive destruction of articular cartilage and subchondral bones^1^. Synovial fibroblasts are thought to play an important role in the pathogenesis of joint destruction in arthritic joints, as excessive inflammatory factors are produced and secreted by rheumatoid arthritis synovial fibroblasts (RASFs)^2,3^. As cell proliferation is a common histological feature of RA, cell cycle regulators may also contribute to RA pathogenesis^4–6^.

The E2F family of transcription factors plays a critical role in the regulation of the G1–S transition and can be subdivided into repressors and activators^7^. Repressor E2Fs (such as E2F5) occupy E2F target promoters during quiescence and in early G1 phase. As cells enter late G1 phase, repressor E2F complexes are replaced by activator E2Fs (such as E2F transcription factor 2; E2F2) that have been released from retinoblastoma protein (Rb) after its phosphorylation^8–10^. E2F transcription factors regulate the expression of numerous genes needed for cell cycle entry and DNA synthesis^11^. A microarray study in peripheral blood mononuclear cells from RA patients found that a significant number of RA-associated genes contain E2F-binding motifs in their promoters, suggesting that proliferation-regulating transcription factors may play a role in RA pathogenesis^12,13^. It has also been reported that abnormal cell differentiation, apoptosis, and tissue development in osteosarcoma and glioma are associated with RA^14,15^.

Previous studies have demonstrated that inhibition of E2Fs by an E2F decoy, oligodeoxynucleotides (ODN), prevents cartilage destruction by inhibition of synovial cell proliferation^16^. E2Fs comprise a family of transcription activators that exert both transcription-activating effects, such as E2F1, E2F2, and E2F3, and transcription-suppressing effects, such as E2F4, E2F5, and E2F6. Our previous microarray analysis found that E2F2 is overexpressed in RA synovial tissues and is detectable in many cells at the sublining region^17^. These findings imply a relationship between E2F2 and RA.

However, to date, no studies have evaluated the precise functions of each of the E2F family members, nor has the mechanism of E2F2 involvement in RA been elucidated. Although suppression of E2Fs is known to ameliorate the inflamed phenotype of RA^2^, the exact function of its members remains obscure. In this study, we focused on the expression and biological activity of E2F2 in RA tissues to further characterize the pathogenesis of RA.

## Results

### E2F2 is overexpressed in the synovial tissue of patients with RA

**Figure 1:**
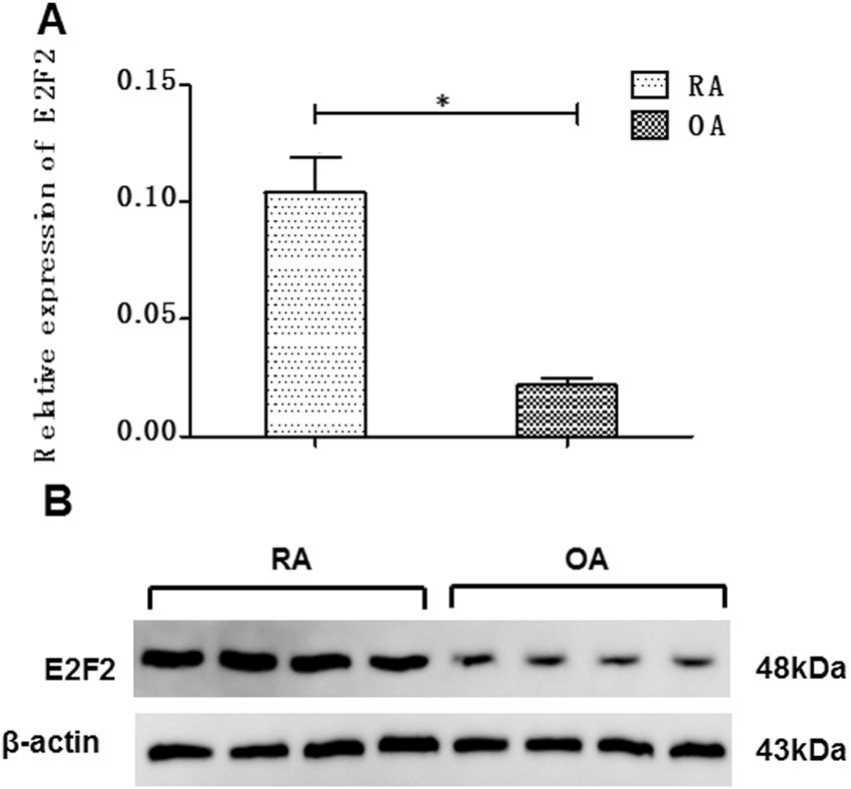
Expression of E2F2 in synovial tissues (STs). **A**, E2F2 mRNA expression in ST from patients with rheumatoid arthritis (RA; n = 10) and osteoarthritis (OA; n = 10) was determined by real-time PCR and calculated as 2−∆∆Ct, with the GAPDH gene used as an endogenous control. **B**, Expression of the E2F2 protein in ST from RA(n = 10) and OA(n = 10) was evaluated by western blot analysis, with the β-actin protein used as a loading control. Values are expressed as mean ± SD of the mean and at least 3 independent experiments were performed; *p < 0.05.

Our previous microarray study demonstrated that E2F2 was significantly more highly expressed in the synovial tissues from RA than in Osteoarthritis (OA)^17^. In this study, we re-analyzed its expression levels in the synovial fibroblasts from RA and OA. Consistently, RT-qPCR (Fig. 1A) and western blot (Fig. 1B) analysis showed increased expression of E2F2 in RASFs at both the RNA and protein level compared to OA.

### Silencing E2F2 suppresses the proliferation, migration, and invasion of RASFs ***in vitro***

**Figure 2:**
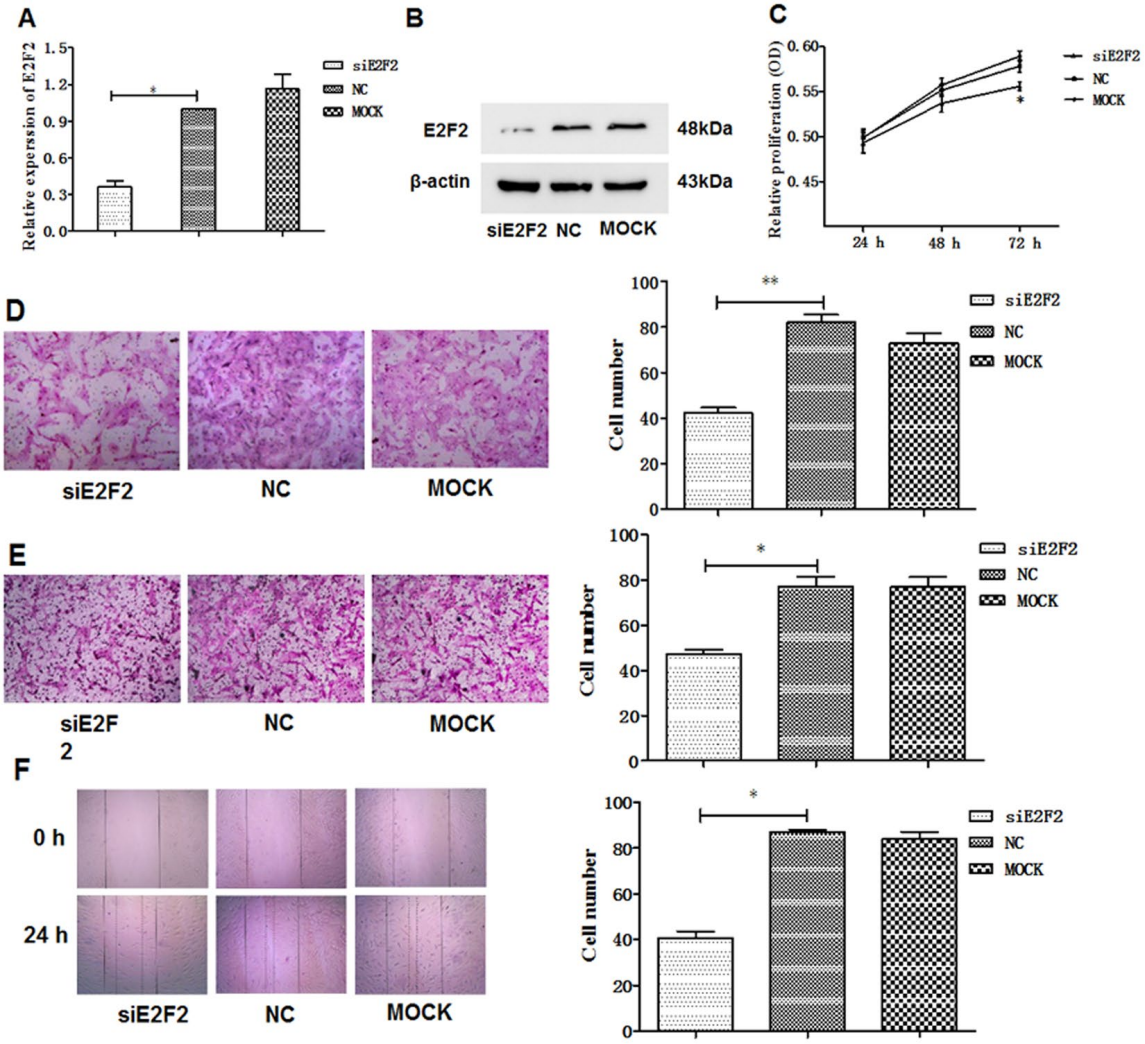
Effect of E2F2 on RASFs proliferation, invasion, migration. **A-B**, RASFs were transiently transfected with siRNA targeting E2F2.Resultant expression was detected using qPCR (**A**) and western blot (**B**). The E2F2 mRNA level and protein levels were both normalized to β-actin. (**C)**, MTS assay was used to assess cell proliferation. (**D**), The invasive ability was performed by transwell assay and the average number of cells that invaded through the filter was quantified. **E-F**, Migration capacity of RASFs 24 h after silencing E2F2 by transwell(**E**) and scratching(**F**). Results of statistical analyses of three independent determinations. NC: negative control; MOCK: transfection-reagent control. Values are expressed as mean ± SD of the mean and at least 3 independent experiments were performed; *p < 0.05.

Since E2F2 was confirmed to be overexpressed in RASFs, we supposed that it is likely involved in mediating the pathological activity of RASFs in disease onset or the progression of RA. To detect the biological activity of E2F2 in RASFs, siRNA targeting E2F2 was designed and synthesized. The inhibition efficiency was showed in Fig. 2A,B.

MTS, transwell, and wound-healing assays were used to assess the effects of silencing E2F2 on the vitality, invasion, and migration capabilities of RASFs. Silencing E2F2 was found to depress cell vitality and the most obvious effect appeared at about 72 h (Fig. 2C). Cells were found to invade the lower chamber in the siE2F2-transfected group significantly less than the respective control groups, as evidenced by Transwell apparatus assay (Fig. 2D). Furthermore, the migration (Fig. 2E) and wound-healing test (Fig. 2F) showed that the number of migrated RASFs was suppressed after E2F2 silencing. These data imply that E2F2 is involved in the disease progression of RA and may influence the biological activity of RASFs.

### E2F2 is sensitive to pro-inflammatory stimulation through different pathways and NF-κB can regulate E2F2 expression by binding to its promoter

To analyze the influence of E2F2 in the immune response, RASFs were treated with well-known pro-inflammatory factors in RA, including IL-6, TNF-α, and LPS. Expression of E2F2 in RASFs with and without stimulation of these factors was analyzed using RT-qPCR and western blot. We found that IL-6, TNF-α, and LPS could all induce the expression of E2F2 in a dose and time dependent manner. 50 ng/ml of IL-6, 50 ng/ml of TNF-α, or 50 ng/ml of LPS induced maximal E2F2 levels at 24 h (data not shown). These data indicate that E2F2 is likely involved in the promotion of RA progression under inflammatory conditions.

**Figure 3:**
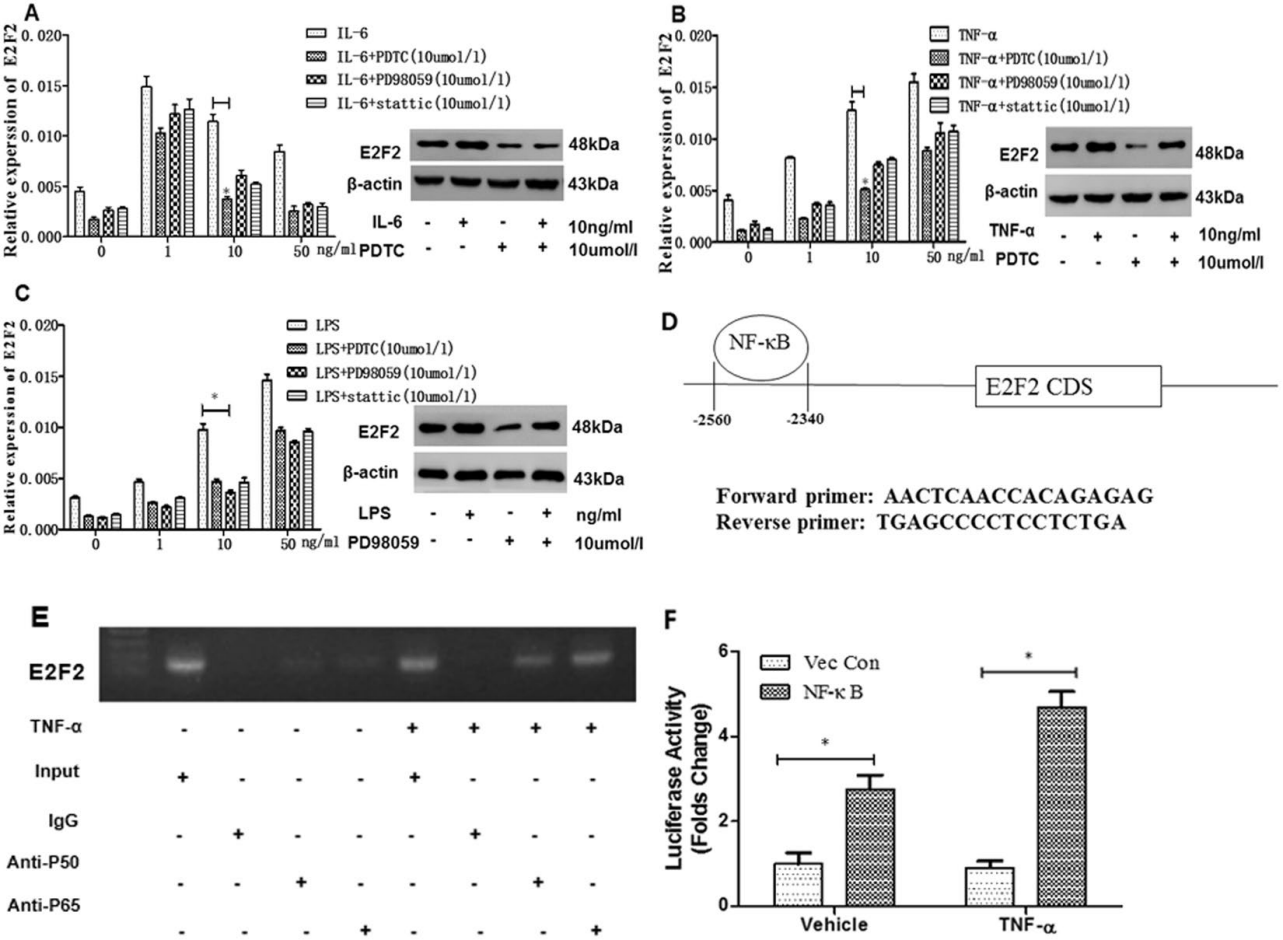
Effects of cytokines on expression of E2F2 and signaling pathways involved in the stimulation. **A–C**, Effect of signaling pathway inhibitors on cytokines induction of E2F2. RASFs were treated for 2 hours with IL-6(**A**), TNF-α (**B**), and LPS(**C**) separately, or in combination with pyrrolidine dithiocarbamate (PDTC), PD98059, or Stattic respectively for 24 h. mRNA and protein expression of E2F2 in cell culture were evaluated by RT-qPCR and western blot respectively. (**D**), Schematic representation of E2F2 promoters, primers for the ChIP assay, NF-кB binding motif in E2F2 promoter. (**E**), Binding of the E2F2 gene promoter in RASFs *in vitro*. ChIP-PCR was used to reveal whether p65 of NF-κB could bind to the promoter of the E2F2 gene in RASFs *in vitro*, and the effect of TNF-α stimulation on the binding was assayed using immunoprecipitated chromatin with anti-p65 antibody; (Input, chromatin input before immunoprecipitation; IgG, immunoprecipitated chromatin with control IgG;Anti-p50, immunoprecipitated chromatin with anti-p50 antibody; Anti-p65, immunoprecipitated chromatin with anti-p65 antibody). (**F**), HEK293T cells stimulated with TNF-α, the luciferase reporter activity of NF-κB was then monitored. The data shown are means ± s.e.m and representation of six independent experiments. *P < 0.05, **P < 0.01, Values are expressed as mean ± SD of the mean and at least 3 independent experiments were performed.

To further uncover the mechanism of E2F2 regulation, we used known inhibitors of several pathways (NF-кB, ERK, and STAT3) associated with inflammatory factors together with IL-6, TNF-α, and LPS, respectively. The results of qRT-PCR and western showed that the induction of E2F2 in response to IL-6, TNF-α, and LPS could be significantly attenuated by PDTC and PD98059, respectively (Fig. 3A,B, and C). These results suggest that all of IL-6, TNF-α, and LPS can regulate expression of E2F2 in RASFs at mRNA level via NF-κB, ERK, and STAT3 pathway. For IL-6 and TNF-α, regulation via NF-κB pathway is the most obvious, while for LPS, regulation via the ERK pathway is the most obvious.

As NF-κB signaling was account for the increase expression of E2F2, especially in response to various stinuli we next performed ChIP and luciferase reporter activity assay to investigate whether NF-κB could directly bind to its promoter region. The schematic representation of NF-κB and promoters of E2F2 was shows as Fig. 3D. The results of ChIP analysis showed that p65 could directly bind to the promoter of E2F2 gene. Importantly, the recruitment of p65 to the E2F2 promoter increased significantly after the stimulation of proinflammatory factor, such as TNF-α (Fig. 3E). The luciferase acticity of E2F2 promoter was measured in HEK293T cells after co-transfection with pcDNA3.1- NF-κB, pGL3-E2F2, and phRL-TK. The data demonstrated that transfection with NF-κB (p50) increased the luciferase activity of E2F2 gene promoter. Furthermore, the above increase was further upregulated after treatment with TNF-α (Fig. 3F).

### E2F2 nuclear translocation is facilitated by extracellular stimuli and E2F2 regulates IL-6 expression by binding to its promoter

**Figure 4:**
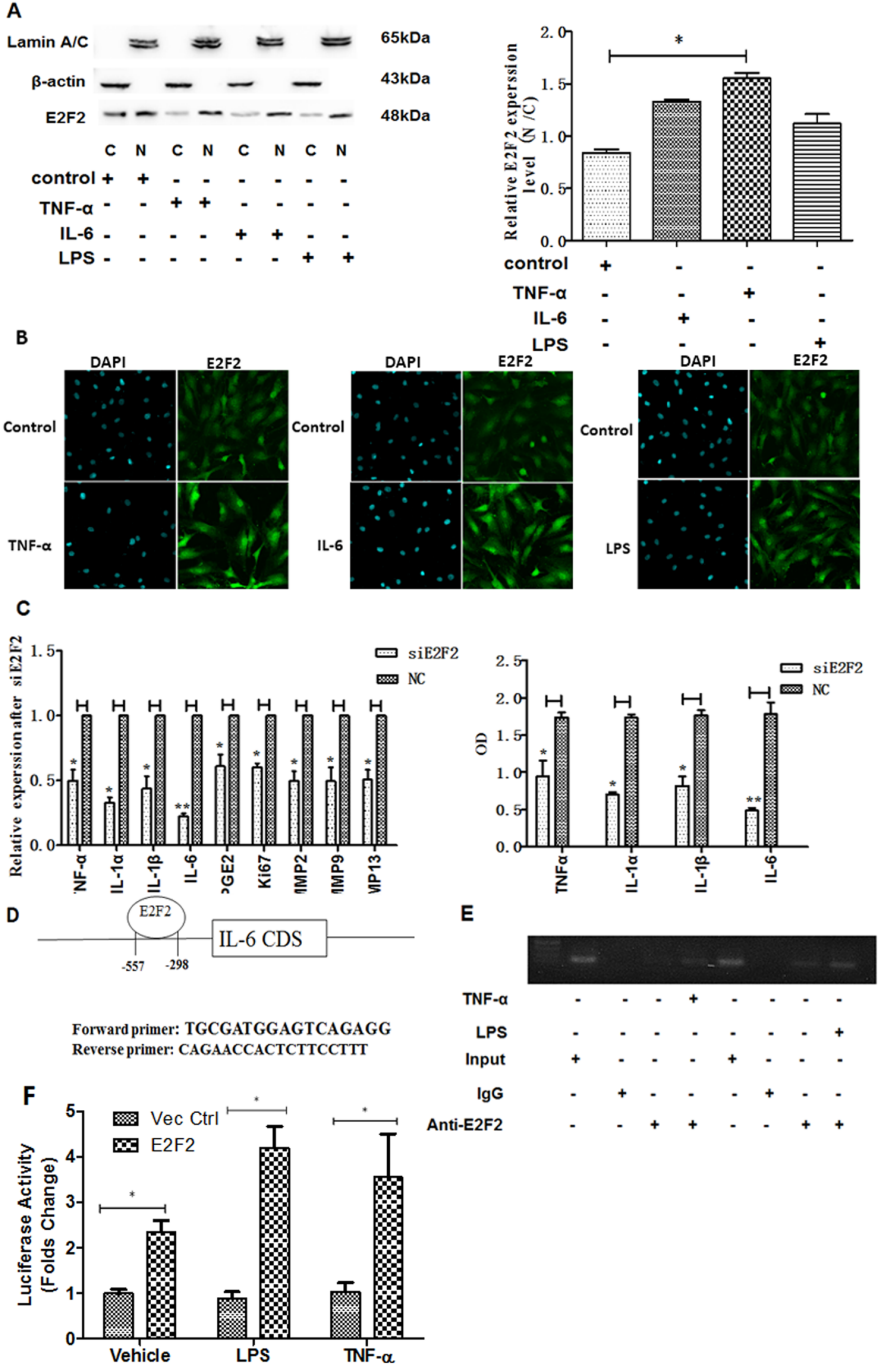
Effect of cytokines on E2F2 nuclear translocation in RASFs and subsequent effects of nuclear translocation. (**A**), Nuclear and cytoplasmic proteins were fractionally extracted from RASFs stimulated with IL-6, TNF-α, and LPS for 6 h. Effects of cytokines on nuclear translocation of E2F2 were determined by Western blot. (Lamin A/C: reference for nuclear extraction; β-actin: reference for cytoplasmic extraction; control: C: Cytoplasmic extraction; N: Nuclear Extraction). (**B**), Nuclear translocation of E2F2 observed using confocal fluorescences microscopy. E2F2 was detected using anti-E2F2 antibody, and Alexa Fluor 488-conjugated IgG. Nuclei were stained with 4′,6′-diamidino-2-phenylinndole (DAPI, blue). The images showed more E2F2 translocation into the nucleus after stimulation with LPS (I), TNF-α(II), and IL-6 (III). (**C**), Measurement of cytokine levels affected by E2F2. mRNA expression and secretion of IL-6, IL-1α, IL-1β, and TNF-α was determined by real-time PCR and ELISA respectively. (**D**), Schematic representation of IL-6 promoters, primers for the ChIP assay, E2F2 binding motif in IL-6 promoter. (**E**), ChIP-PCR was used to determine whether E2F2 affect IL-6 secretion by binding to its promoter directly. Effects of TNF-α and LPS on the bindation between E2F2 and the promoter of IL-6 were also investigated. (Input, chromatin input before immunoprecipitation; Anti-E2F2: immunoprecipitated chromatin with anti-E2F2 antibody; IgG, immunoprecipitated chromatin with control IgG.) **(F)**, HEK293T cells stimulated with LPS and TNF-α, the luciferase reporter activity of E2F2 was then monitored. Values are expressed as mean ± SD of the mean and at least 3 independent experiments were performed. *P < 0.05, **P < 0.01.

As E2F2 is a transcriptive factor, nuclear translocation is pivotal for its function. We performed western blotting to detect its expression both in the cytoplasmic and nuclear compartments and immunofluorescence to observe effect of extracellular stimuli on nuclear transcolation. E2F2 protein was detected in both the cytoplasm and nucleus (Fig. 4A) even without any stimulation. Interestingly, when RASFs were stimulated by extracellular LPS, IL-6, and TNF-α, more nuclear translocation was observed both from western blotting (Fig. 4A) and from immunofluorescence (Fig. 4B). Notably, the tendency of E2F2 nuclear translocation was more obvious in the presence of IL-6.

As a result of more nulcear translocation, E2F2 can regulate the expression of numerous genes because it’s an important transcription factor^18,19^. To gain further insight into the role of E2F2 in the pathology of RA, we chose genes known to be involved with RA pathology, including ki67, a marker to measure the proliferative state of RASFs^20^, cytokines such as TNF-α, IL-1α, IL-1β, IL-6, PGE2^21^, and matrix metalloproteinases which can degrade extracellular matrix (ECM), including MMP2, MMP9, and MMP13, which in turn lead to the invasion of RASFs to adjacent bone or cartilage^22^.qRT-qPCR was used to test the expression of these genes. The data showed that the expression of these nine gene was suppressed in E2F2 knocked-down RASFs. Besides effects on downstream genes expression, we supposed that E2F2 may be involved in the inflammatory exacerbation of RA. To test our supposition, we investigated whether E2F2 has effect on production of cytokines by assessing the expression of IL-6, IL-1α, IL-1β and TNF-α using ELISA. Consistent with our hypothesis, real-time PCR found E2F2 silencing to inhibit the mRNA expression of IL-6, IL-1α, IL-1β, and TNF-α in RASFs (Fig. 4C left). Moreover, ELISA results showed that the secretion of IL-6, IL-1α, IL-1β, and TNF-α could also be suppressed in the presence of siRNA transfection targeting E2F2 compared to controls (Fig. 4C right).

As IL-6 had the most striking effect, ChIP analysis was applied to further characterize the exact mechanism of E2F2 action on IL-6. The schematic representation of E2F2 binding motif and promoter of IL-6 was showed in Fig. 4D. Our ChIP results found that E2F2 could bind to the promoter of IL-6 gene. Of interest, the binding of E2F2 to the IL-6 promoter increased significantly in the presence of TNF-α and LPS stimulation (Fig. 4E). We also applied luciferase reporter gene assay to further confirm the regulation of E2F2 towards IL-6 promoter. The data showed that the luciferase activity of IL-6 promoter increased after overexpressing E2F2. Consistent with that results of ChIP analysis, its luciferase activities were further upregulated with the stimulation of LPS and TNF-α (Fig. 4F).

### Production of IL-6 by E2F2 in RASFs regulates RASF sensitivity to extracellular stimuli

**Figure 5:**
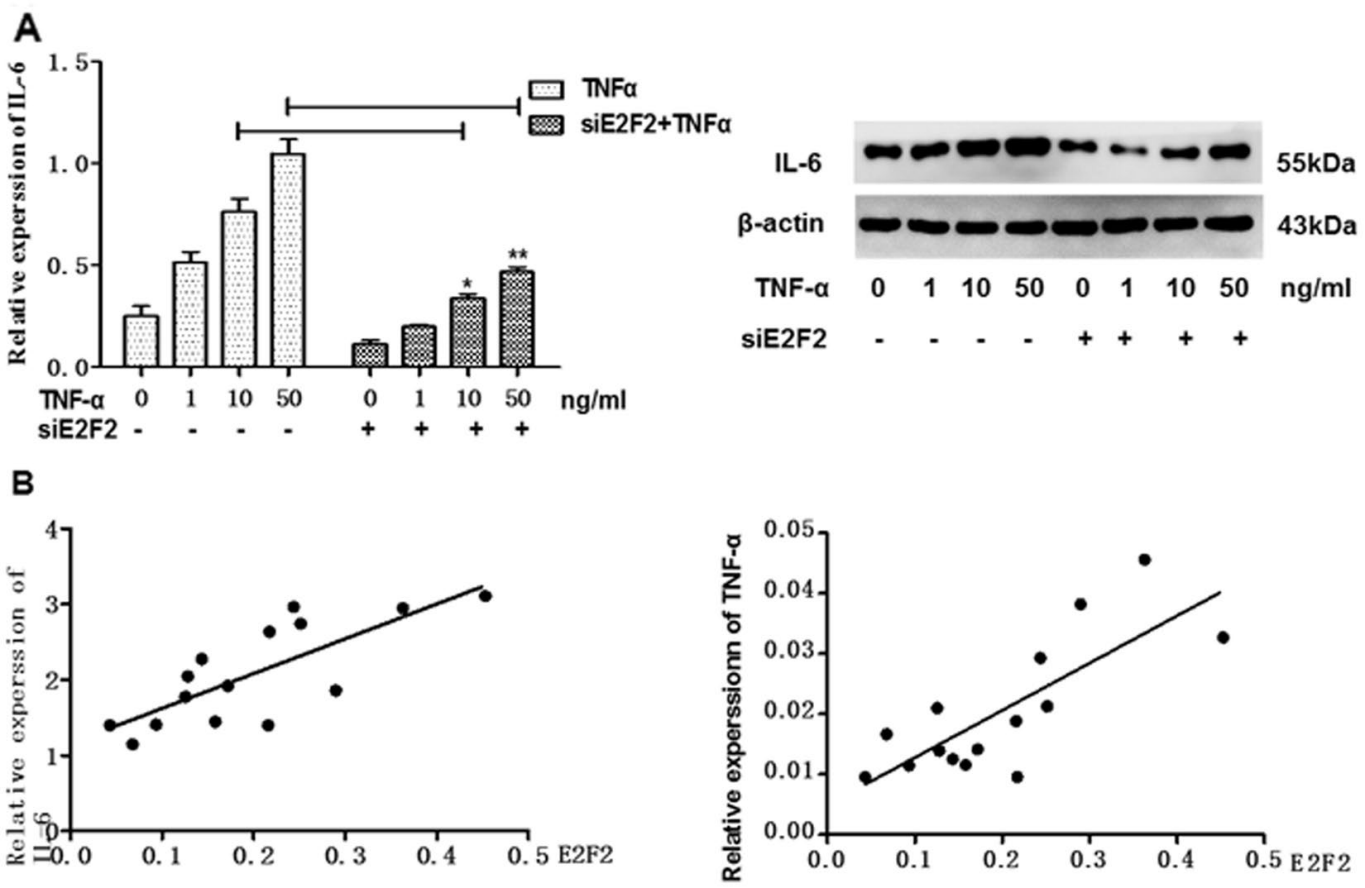
Effect of E2F2 on IL-6 expression and correlations between serum E2F2 and cytokine levels in patients with rheumatoid arthritis. (**A**), Effect of siE2F2 on TNF-α stimulated IL-6 expression detected by RT-qPCR (left) and Western blot (right). (**B**), Correlations between mRNA levels of E2F2 and IL-6 or TNF-α secreted into the supernatant of RASFs. qRT-PCR was used to quantify mRNA levels of E_2_F_2_, and ELISA was performed to quantify the levels and IL-6 or TNF-α respectively secreted into the supernatant of RASFs(n = 15). Pearson correlation analysis was used to assay the correlations. Values are expressed as mean ± SD of the mean and at least 3 independent experiments were performed. *P < 0.05. **P < 0.01.

TNF-α is an important cytokine that stimulates RASFs and inflammatory cells to aggravate synovial inflammation^3^. To determine whether E2F2 regulates the biological activity of RASFs in response to TNF-α stimulation, the production of the proinflammatory cytokine IL-6 was analyzed in TNF-α stimulated RASFs with or without siE2F2. As expected, TNF-α could up-regulate the expression of IL-6 in RASFs. However, stimulation of IL-6 by TNF-α was suppressed after silencing E2F2 in RASFs (Fig. 5A).

To explore the relationship between E2F2 and IL-6 or TNF-α in RA patients, we analyzed levels of IL-6 and TNF-α in supernatant of RASFs (n = 15) respectively using ELISA. At the same time, mRNA level of E2F2 expressed in RASFs of the same cohort was also assayed using qRT-PCR. Significant correlation was found between E2F2 and IL-6 levels (r^2^ = 0.5940, P = 0.0008, Fig. 5B left) and TNF-α (r^2^ = 0.6142, P = 0.0005, Fig. 5B right). These results further support the pro-inflammatory mediation role of E2F2 in RA.

## Discussion

Previous studies have shown that inhibition of E2Fs by ODN can prevent cartilage destruction by inhibition of synovial cell proliferation^2^. Our previous microarray assay found E2F2 to be overexpressed in synovial tissue of RA^17^. Following studies have also evaluated the expression of E2F2 in the synovial tissues from RA and OA patients and found that they are significantly higher-experssed in RA synovial tissue than in OA^15^. In this study, markedly higher E2F2 expression has also been found in RASFs than in OASFs, further supporting its likely involvement in the pathogenesis of RA disease^15^.

E2F2 is a member of the E2F family of transcription factors and binds DNA cooperatively with DPDP1-polypeptide through the E2 recognition site in the promoter region of genes to produce products involved in cell cycle regulation or DNA replication^2,16,17^. RA, a chronic inflammatory disease, is characterized by hyperplasia of the synovial fibroblasts, which is due in part to increased cell proliferation. In our study, silencing E2F2 resulted in the inhibition of proliferation of RASFs, along with the suppression of the expression of Ki67, a marker of proliferative ability of RASFs. Silencing E2F2 also resulted in suppression of invasion of RASFs, along with matrix metalloproteinases, which can degrade extracellular matrix (ECM), including MMP2, MMP9, and MMP13, which in turn lead to the invasion of RASFs to adjacent bone or cartilage. All these results demonstrate that E2F2 is biologically functional in RA and its active form in RASFs could accelerate the progression of RA. The results were consistent with previous studies about the function of E2F2 in various tumors^23^.

Previous studies have shown that IL-6 and TNF-α are important cytokines involved in the pathogenesis of RA^21,24^. Moreover, bacterial and viral infections are associated with the occurrence and pathogenesis of reactions in RA, and LPS is well recognized as a stimulating factor in RA inflammation^25^. Our observation that E2F2 has an impact on RASFs led us to explore the possibility that E2F2 might be functionally linked to these inflammatory factors. Indeed, we found that IL-6, TNF-α, and LPS are all responsible for induction of the expression of E2F2 in RASFs. Previous studies have shown that cytokines could activate the signaling pathways, such as NF-кB and ERK, in RASFs^26^. To investigate which pathways are account for the up-regulation of E2F2 expression in RASFs, we used specific inhibitors to evaluate the involvement of the pathways. Our results suggest that all of IL-6, TNF-α, and LPS can regulate expression of E2F2 in RASFs at mRNA level via NF-κB, ERK, and STAT3 pathway. For IL-6 and TNF-α, regulation via NF-κB pathway is the most obvious, while for LPS, regulation via the ERK pathway is the most obvious.

More importantly, the dynamic recruitment of NF-кB on the E2F2 promoter implies that E2F2 was an important convergent point of RASFs in response to inflammatory stimuli.

Pro-inflammatory cytokines, including IL-1α, IL-1β, IL-6, and TNF-α, are expressed by various cell types in inflamed synovium^21,27^. We also demonstrated that E2F2 is involved in the inflammatory response in RA, as evidenced by the fact that silencing E2F2 could suppress levels of IL-1α, IL-1β, and IL-6. Importantly, positive correlations were identified between E2F2 expression and the production of IL-6 and TNF-α in the serum of RA patients. These data showed multiple levels of interaction between E2F2 and cytokines *in vitro*, implying E2F2 as a potential treatment target for RA.

In RA, RASFs are hyper-responsive to immune stimulation. When activated by proinflammatory mediators like IL-6, RASFs produce growth factors and cytokines that stimulate extracellular matrix deposition, a pathological characteristic that is increased in RA^28,29^. Cytokines and other stimuli could induce the activation of the E2F family of transcription factors, which translocate into the nucleus to transcribe a variety of genes involved in inflammation, immune regulation, and apoptosis^30,31^. In our study, E2F2 protein was detected in both cytoplasm and nucleus. More interestingly, nuclear translocation of E2F2 was facilitated by extracellular stimulation from extracellular stimuli, especially IL-6. The increased enrollment of E2F2 on the promoter of IL-6 gene indicates the existence of positive loop between E2F2 and IL-6. Furthermore, correlation between E2F2 and IL-6 in serum of RA patients, and suppression of IL-6 expression in RASFs by silencing of E2F2 strongly confirmed that E2F2 may function as a pro-inflammatory factor in RA.

In summary, this study demonstrated that the increased expression of E2F2 in RA synovial tissues contributes to the abnormal proliferation, invasion and cytokine production of RASFs. The induction of E2F2 in RASFs under inflamed condition further confirmed the pro-infalmmatory role of E2F2 in RA. Of note, a positive feedback loop among NF-кB/E2F2/IL-6 may exist in RASFs, finally leading to the hyper-inflammation of RA. Totally, our finding indicates E2F2 to be a potential target in the therapeutic approach of RA. Further studies focusing on E2F2 may highlight more pathological mechanisms of RA.

## Materials and Methods

### Sample collection

Synovial tissue and fluid samples were collected during knee joint replacement surgery from patients with RA (n = 15, 9 female, age 29 to 72 years old, mean 51 years) and patients with Osteoarthritis (OA, n = 15, 7 female, age 39 to 77 years old, mean 62 years). All of the patients fulfilled the American College of Rheumatology (ACR) diagnosis criteria for RA and OA. Demographic characteristics are presented in Supplementary Table S1. Participants provided written informed consent to participate in the study and to allow their biological samples to be genetically analyzed. The Ethical Committee of the Shandong Academy of Medicinal Sciences approved this study and the approval number is 2014-19. The study was conducted in compliance with the Helsinki Agreement or with the research ethics standards of the country of origin of the report. The information of patients adopted in this article was included in Table 1.

**Table 1:** Demographic characteristic of studied pupulations.

Sample	Characteristics	Control (OA)	RA
Synovium/Fluid	Number of subjects	15	15
	Female/Male	8/7	9/6
	Age	39 to 77 (mean 62)	29 to 72(mean 51)
	CRP	—	32–298 mg/L(mean 73 mg/L)
	anti-CCP	—	28–458 u/mL(mean 279.6 u/mL)
	RF	—	38–316 u/mL(mean 183.2 u/mL)

### Culture and identification of RASFs

RA synovial tissue was finely chopped and incubated with type II collagenase (1 mg/mL, Sigma-Aldrich) in Dulbecco’s modified Eagle medium (DMEM, HyClone, Thermo Scientific) for 6 h at 37 °C, with 5% CO2 (Thermo Scientific). The tissue was treated with 0.25% trypsin (Solabio) diluted in phosphate buffered saline (PBS) solution at a volume equivalent to the DMEM. Cells were filtered and cultured overnight in DMEM, supplemented with 10% fetal bovine serum (FBS, HyClone, Thermo Scientific), penicillin (100 IU/ml), and streptomycin (100 µg/ml, Gibco) for three passages. RASFs at passage 4–6 were used for the study, which were negative for CD14, CD3, CD19 and CD56 expression as identified by flow cytometry analyses, were used for the study.

### Inhibition of E2F2 expression with small interfering RNA (siRNA)

Three siRNA targeting E2F2 were designed and synthesized by QIAGEN. The most efficiency siRNA was used in the following study. The sequence is 5′-TCCGTGCTGTTGCAACTTTA-3′. Cultured RASFs were transfected with siRNA at 100 nmol/l using a HiPerFect transfection reagent (QIAGEN, Germany) according to the manufacturer’s protocol. The cells were harvested foranalysis 24 h following the transfection. A negative siRNA (5′-UUCUCCGAACGUGUCACG UTT-3′) was used as the negative control and reagent-only transfection was used as mock treatment.

### Quantitative real-time PCR (RT-qPCR)

Total RNA was extracted from the cultured cells and the human tissue using Total RNA Kit (OMEGA) and reverse-transcribed using a ReverTra Ace qPCR RT Kit (Toyobo, Japan) according to the manufacturer’s protocol. qPCR was conducted using the LightCycler 480 (Roche, Germany) using the following amplification protocol: denaturation at 95 °C for 10 minutes, then 40 cycles of denaturation at 95 °C for 10 seconds followed by annealing at 60 °C for 1 minute and extension at 72 °C for 1 second. The comparative threshold cycle (Ct) method was used to analyze the relative expression of mRNA. The relative target gene expression was normalized relative to glyceraldehyds-3-phosphate dehydrogenase (GAPDH) mRNA levels. Primers for qPCR were also designed accordting to the consensus sequence. Primers for RT-qPCR were also designed according to the consensus sequence. All mRNA transcripts of E2F2 were abstracted from Genbank: NM_004091.3, XM_005245748.3, XM_005245749.3, XM_011540868.2, XM_011540870.2, and XM_011540871.2. The consensus sequence was determined using Align X function of the software Vector NTI Advance 11(Invitrogen Inc. U.S.A). The primers were designed according to the consesus sequenece using the software LightCycler Probe Desigh Software 2.0 (Roche Inc.,Switzerland) as follows: 5′-CCTTGGAGGCTACTGACAGC-3′ (forword) and 5′-CCACAGGTAGTCGTCCTGGT-3′ (reverse). Primers of the reference gene GAPDH were as follows: 5′-CACCATCTTCCAGGAGC-3′ (forword) and 5′-AGTGGACTCCACGACGTA-3′ (reverse).

### Western blotting

Tissue samples of RA and OA patients and the cultured cells were homogenized in RIPA lysis buffer (Beyotime, China) with protease inhibitors on ice for 30 min to extract the protein. The preparation was centrifuged at 14000 rpm for 30 min at 4 °C. The protein concentration was measured with a Bio-Rad protein assay system (Bio-Rad,USA). Twenty-five micrograms of the total protein were fractionated by sodium dodecyl sulfate-polyacrylamide gel electrophoresis (SDS-PAGE), transferred to a PVDF membrane (Amersham, USA) with wet transfer and blocked in 10 mmol/L Tris-buffered saline (TBS) containing 0.1% (v/v) Tween-20 (TBST) and 5% (w/v) nonfat milk, then immunoblotted with specific primary antibodies. Anti-human E2F2 antibody was diluted at 1:1000 (Millipore, USA), and anti-human β-actin antibody diluted at 1:1000 (Beoytime, China).

### Cell proliferation assay

RASFs were seeded into 96-cell plates at 2–4 × 10^4^ cells per well and transfected with siRNA (100 nmol/l) as described above. 20 μl of [3-(4,5-dimethylthiazol-2-yl)-5-(3-carboxymethoxyphenyl)-2-(4-sulfophenyl)-2H- tetrazolium (MTS) solution was added after incubation for 24 h, 48 h and 72 h, and samples were incubated at 37 °C in the dark for another 1 h. Absorbance at 490 nm was measured by multi-plate reader (Perlong, China).

### Cell invasion, and migration assay

Cell invasion ability was measured in the transwell apparatus (Becton Dickinson, USA), wound-healing assay was performed to test the migration ability of RASFs, following procedures described previously^18^.

### Expression of E2F2 regulated by cytokines

RASFs were seeded into 24-well plates (density 2–4 × 104/well) Then cells were cultrued for 12, 24, 48 h in the presence of 0, 1, 10, 50 ng/ml of interleukin 6 (IL-6), tumor necrosis factor alpha (TNF-α), or lipopolysaccharide (LPS) respectively, or combined with 10μmol/l of PDTC (an inhibitor of nuclear transcription factor kappa B (NF-кB), Abcam, ab141406), PD98059 (an inhibitor of extracellular regulated protein kinases (ERK-1/2), Abcam, ab120234), or Static (an inhibitor of signal transducers and activators of transcription (STAT3), Abcam, ab120952), respectively. For the siRNA blocking assay, 0, 1, 10, 50 ng/ml of TNF-α with or without 100 nmol/l siRNA-E2F2 were added to seeded RASFs for 24 h (see below).

### Probing of signaling pathways involved in E2F2 induction

Special inhibitors of the RASFs were seeded into 12-well plates (density 1 × 10^5^/well). Confluent cells were starved by decreasing serum to 2% in medium for 24 h. The starved RASFS were stimulated for 12 h with IL-17 (50ng/ml) and signal pathway inhibitor (PDTC, PD98059, Stattic). Expression of E2F2 was detected by real-time PCR and Western blotting.

### Chromatin immunoprecipitation assay (ChIP)

RASFs were stimulated with 10 ng/ml of TNF-α and LPS separately for 24 h or left unstimulated. Chromatin from RASFs was fixed and immunoprecipitated using the ChIP assay kit (Millipore, USA) following the manufacturer’s instructions. One-tenth of purified chromatin without antibody was used as input control. Other purified chromatin was immunoprecipitated using 2 ng of anti-E2F2 (Milipore, USA), anti-p50 (Cell signaling, USA), anti-p65 (Cell signaling,USA) antibodies, or an irrelevant antibody control (anti-IgG, Santa Cruz, USA). After DNA purification, the presence of the selected DNA sequence was assayed using RT-qPCR. The primers used are as follows: E2F2: 5′-CTCAGATGACTGGTAGTA-3′(forword) and 5′-TCAGTGACCAGATTAA CA-3′ (reverse), IL-6: 5′-CCTTGGAGGCTACTGACAGC-3′(forword) and 5′-CCACAGGTAGTCGTCCTGGT-3′(reverse). The fold enrichment of immunoprecipitated samples was normalized to input and expressed relative to controls (IgG).

### Extraction of cell nucleus protein and cytoplasmic protein RASFs

RASFs were stimulated with 10 ng/ml of TNF-α, LPS and IL-6 separately for 24 h or left unstimulated. Different component proteins from RASFs was extracted using the ProteoExtract ™ Subcellular Proteome Extraction Kit S-PEK (Calbiochem, USA) following the manufacturer’s instructions. Protein samples were deal with by Western Blot. β-actin antibody and laminA/C antibody were used to verify the success of nuclear separation, laminA/C antibody is the nucleus internal reference. β-actin antibody is the cytoplasmic internal reference. E2F2 antibody is used to detect the E2F2 protein.

### Enzyme-linked immunosorbent assay (ELISA)

The culture medium was collected and centrifuged at 1000 rpm for 5 min at 4 °C. 100 μL medium were added to a 96-well microplate (Costar, USA), which was stored overnight at 4 °C. After gently washing with PBST, 1% bovine serum albumin (BSA) plus 5% sucrose was used for blocking for 1 h at 37 °C. Following three PBS washes, antibodies were applied to the plate for overnight incubation. Plate was washed, blocked, and treated with a 1:2000 dilution of anti-rabbit Ig-G horse radish peroxidase (HRP) antibody (Proteintech, USA) was added, and the plate was incubated for 3 h at 37 °C. Staining was developed by TMB Kit (CWBIO, China). The absorbance at 450 nm was measured with a plate reader (Synergy HT, USA). Antibodies against IL-1α, IL-1β, IL-6, and TNF-α (all from Abcam, USA, diluted 1:1000) were applied in this study.

### Nuclear and cytoplasmic extraction

RASFs were transfected with siRNA (100 nmol/l) for 24 h and then nuclear and cytoplasmic protein was extracted separately using the NE-PER Nuclear and Cytoplasmic Extraction Reagents (Thermo Scientific, USA) per the manufacturer’s instructions.

### Plasmid constructs

The coding regions of NF-κB (p65) or E2F2 were amplified from cDNA of HEK293T cell and cloned into pcDNA3.1 vector. The promoter region of E2F2 or IL-6 gene was amplified from HEK293T and cloned into the pGL3-basic vector using Knp1 and EcoRV restriction sites, respectively.

### Luciferase reporter gene assay

HEK239T cells (2 × 104 cells/well; 96-well plate) were seeded in triplicate in 24-well plates and was transfected with 80ng/well luciferase reporter gene plasmid for IL-6 or E2F2 using Lipofectamin 2000, as described by the manufacturer (Invitrogen). pcDNA3.1-E2F2 or pcDNA3.1- NF-κB were transfected into the above cells as indicated. In all cases, 40ng/well of phRL-TK reporter gene activity was measured using the Dual Luciferase Assay system (Promega). Data are expressed as the mean fold induction ± s.e.m. relative to control levels from a minimum of three separate experiments.

### Immunofluorescence

Cells were seeded in 48 well plate with confluence of 50%. Then cells were fixed with 4% paraformaldehyde for 20 min and permeabilized with 0.1% Triton X-100 for 10 min after indicated treatment. 5% BSA was used for blockage for 1 h at room temperature. Cells were then incubated with primary antibodies against E2F2 (1:200, ab65222, Abcam, USA) at 4 °C overnight. After being washed with PBS three times, cells were incubated with Alexa Fluor 488-conjugated secondary antibodies (1:500, Invitrogen) for 1 h at 37 °C. The cells were mounted with prolong gold antifade reagent with DAPI (Invitrogen) for 10 min. After rinsed with PBS for three times, samples were analyzed with confocal fluorescence microscope (Olympus, Japan).

### Statistical analysis

The statistical significance was performed using Graph Pad Prism version 5 software, USA. The results were expressed as mean ± S.E.M. of 5 different experiments. The date were analysed by two-way analysis of variance (two-way ANOVA) followed by Bonferroni’s multiple comparison test. The statistical significance of difference in the central tendencies was designated as *P < 0.05 and ** P < 0.01.

### Data Availability

All data generated or analysed during this study are included in this published article.

### Ethics approval

Synovial tissues used in this study were provided by the Shandong Provincial Hospital. Tissues were collected during knee replacement surgery from patients with RA and osteoarthritis (OA).Participants provided written informed consent to participate in the study and to allow their biological samples to be genetically analyzed. The Ethical Committee of the Shandong Academy of Medicinal Sciences approved this study.

## Electronic supplementary material

Supplementary figures

Raw data of Fig 3A-C
