# Supplementary material for: A critical role of E2F transcription factor 2 in proinflammatory cytokines-dependent proliferation and invasiveness of fibroblast-like synoviocytes in rheumatoid Arthritis
Source: Sci Rep. 2018 Feb 8;8:2623. doi: 10.1038/s41598-018-20782-7 (PMC5805761; doi:10.1038/s41598-018-20782-7)

**A critical role of E2F transcription factor 2 in proinflammatory cytokines-dependent proliferation and invasiveness of fibroblast-like synoviocytes in rheumatoid Arthritis**

**Rui Zhang^1,2,3^ , Lin Wang^1,2,3^,, Ji-hong Pan^#1,2,3^, Jinxiang Han^#1,2,3^**

#:Co-correspondence authors.

Supplementary Fig 1 Optimal results of concentration and time of proinflammatroy cytokines and pathways inhibitors .


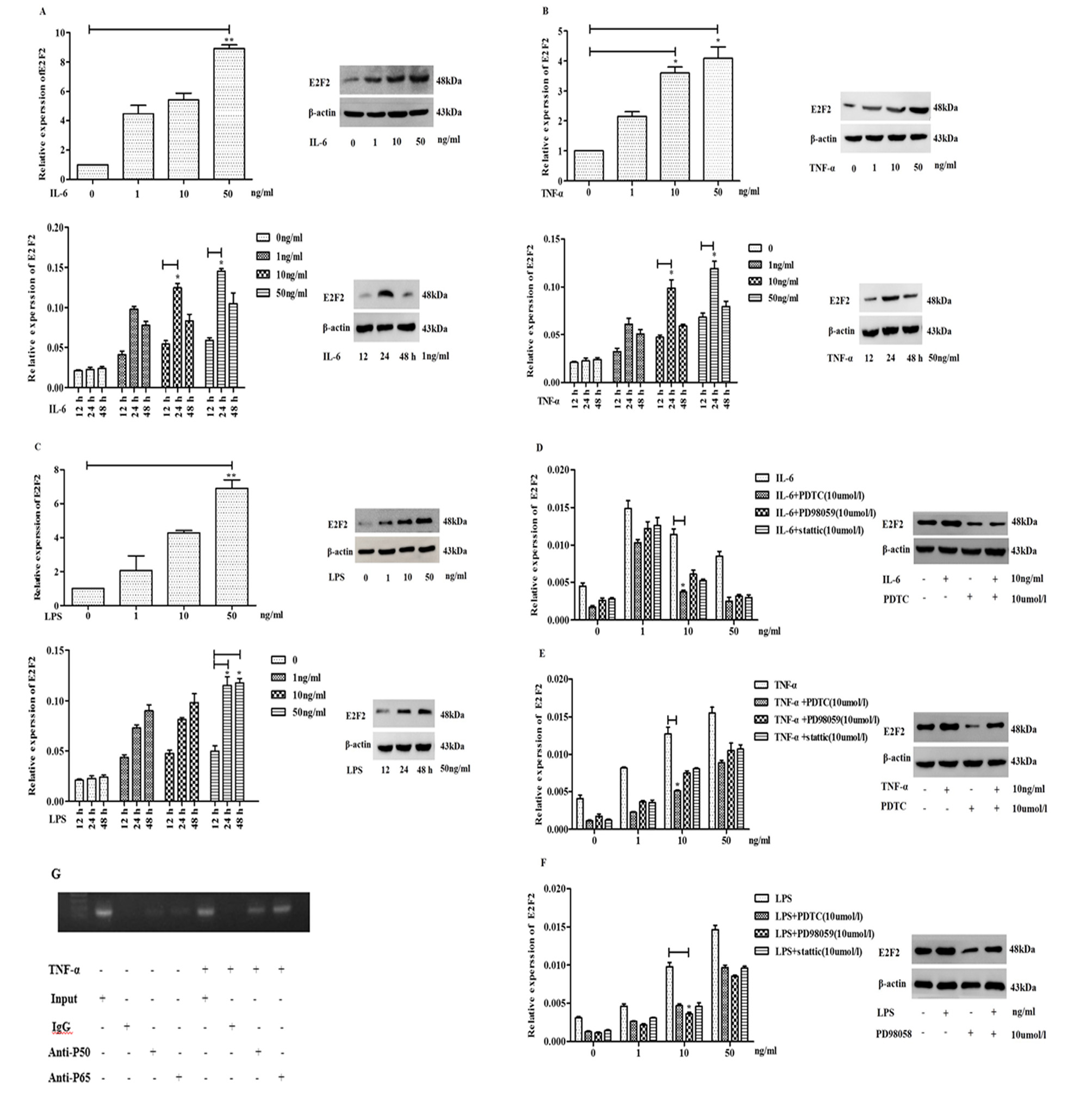


Supplementary Fig 2 Unchopped western blots in manuscripts.


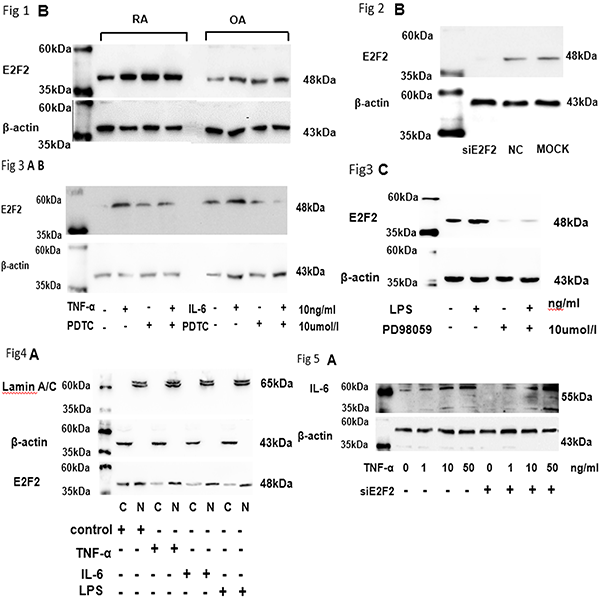


Table 1 Demographic characteristic of studied populations


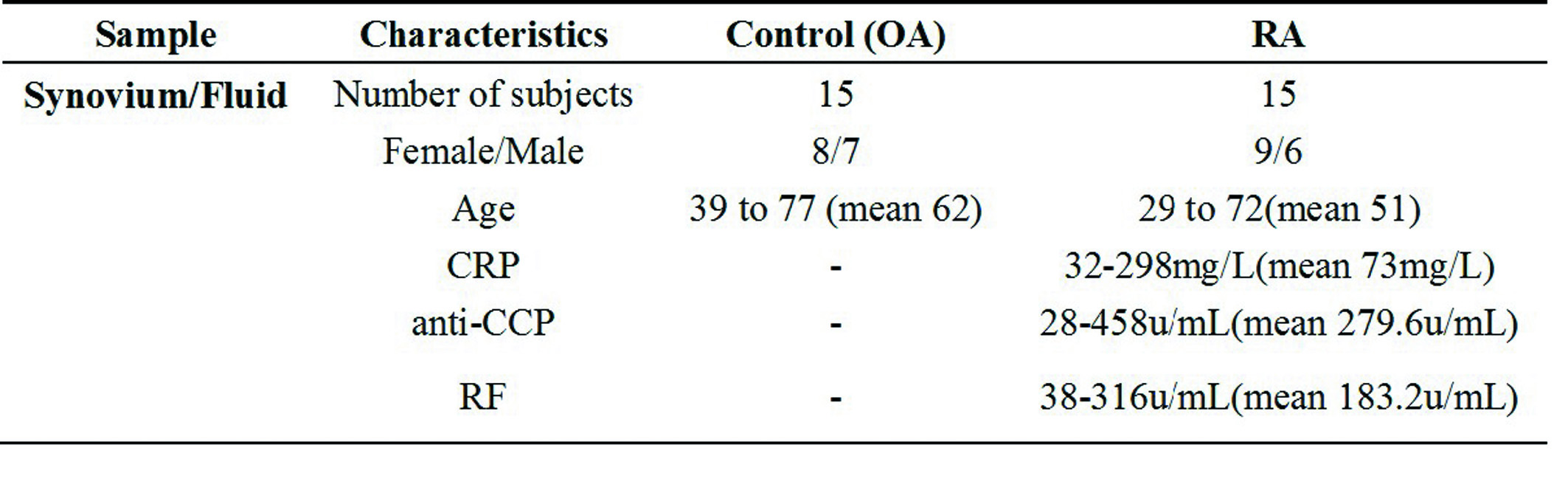

Supplement: Supplementary file 1 — Supplementary figures [file 41598_2018_20782_MOESM1_ESM.doc]
